# Supplementary material for: An ensemble deep learning framework for energy demand forecasting using genetic algorithm-based feature selection
Source: PLoS One. 2025 Jan 15;20(1):e0310465. doi: 10.1371/journal.pone.0310465 (PMC11734974; doi:10.1371/journal.pone.0310465)
Supplement: Attachment — Submitted filename: Response to Reviewers.docx [file pone.0310465.s001.docx]

Replies to Concerns of Editor and Reviewers

**An Ensemble Deep Learning Framework for Energy Demand Forecasting Using Genetic Algorithm-Based Feature Selection**

PONE-D-24-12727

Dear Editors and Reviewers,

We would like to express our sincere gratitude to the editors and reviewers for their thorough examination and insightful feedback on our manuscript.

In response to the reviews, we have thoroughly revised our manuscript, taking into careful consideration every comment and suggestion provided. **Revised portions are highlighted in the manuscript in yellow. The "+" sign indicates that this paragraph has been added in the revised manuscript.** The main corrections in the paper and the responses to the reviewer's comments are listed below.

**Response to the Editor**

**Comment:** *The author have employed stochastic DL models (LSTM, BiLSTM, GRU) and a stochastic method GA. Therefore, it is suggested to repeat the simulations at least 10 times and apply non-parametric statistical tests like Wilcoxon Signed Rank Test on the obtained results to draw decisive conclusions. Otherwise the conclusions might not be reliable.*

**Author Response:** We acknowledge the importance of ensuring the reliability of our conclusions given the stochastic nature of our models and method. To address this, we have conducted the following steps:

1. **Repeatead Simulations:** We have repeated the simulations 10 times for each stochastic deep learning model (LSTM, BiLSTM, GRU) and the genetic algorithm (GA) to ensure the reliability of our results. For each run, we recorded the performance metrics (RMSE, MAPE, MAE) shown in below tables.

**Table 5.** Overall performance metrics across 10 runs on weekdays dataset

| **No of Trails**  **Model Error** | | **1st** | **2nd** | **3^rd^** | **4th** | **5th** | **6th** | **7th** | **8th** | **9th** | **10th** |
| --- | --- | --- | --- | --- | --- | --- | --- | --- | --- | --- | --- |
| **RMSE** | LSTM | 148.3 | 145.2 | 147.9 | 151.8 | 146.8 | 144.98 | 141.8 | 145.3 | 143.7 | 150.6 |
|  | BiLSTM | 138.5 | 141.2 | 139.3 | 137.5 | 134.9 | 133.9 | 139.4 | 132.5 | 137.9 | 136.4 |
|  | GRU | 145.2 | 147.2 | 138.9 | 145.7 | 143.8 | 147.9 | 141.5 | 149.6 | 146.8 | 157.6 |
|  | **En-GA** | 130.8 | 131.5 | 136.4 | 140.8 | 136.9 | 145.2 | 135.6 | 147.6 | 142.8 | 150.6 |
| **MAPE %** | LSTM | 0.42 | 0.42 | 0.41 | 0.43 | 0.41 | 0.44 | 0.43 | 0.45 | 0.42 | 0.41 |
|  | BiLSTM | 0.38 | 0.34 | 0.37 | 0.36 | 0.39 | 0.38 | 0.40 | 0.39 | 0.37 | 0.35 |
|  | GRU | 0.40 | 0.41 | 0.43 | 0.37 | 0.36 | 0.39 | 0.38 | 0.41 | 0.42 | 0.34 |
|  | **En-GA** | 0.35 | 0.36 | 0.39 | 0.34 | 0.33 | 0.35 | 0.37 | 0.39 | 0.41 | 0.32 |
| **MAE** | LSTM | 115.3 | 118.6 | 115.2 | 114.5 | 116.9 | 112.3 | 119.6 | 121.6 | 117.5 | 116.9 |
|  | BiLSTM | 106.8 | 109.9 | 112.5 | 108.6 | 110.5 | 107.6 | 115.2 | 118.8 | 116.3 | 113.2 |
|  | GRU | 107.6 | 108.3 | 111.6 | 105.7 | 109.4 | 105.3 | 112.1 | 113.9 | 115.7 | 110.0 |
|  | **En-GA** | 99.6 | 101.2 | 98.6 | 97.3 | 101.4 | 102.9 | 99.3 | 108.2 | 104.5 | 98.7 |

**Table 6.** Overall performance metrics across 10 runs on on weekend dataset

| **No of Trails**  **Model Error** | | **1st** | **2nd** | **3^rd^** | **4th** | **5th** | **6th** | **7th** | **8th** | **9th** | **10th** |
| --- | --- | --- | --- | --- | --- | --- | --- | --- | --- | --- | --- |
| **RMSE** | LSTM | 180.6 | 182.6 | 185.2 | 184.6 | 181.4 | 196.3 | 189.7 | 184.5 | 180.2 | 186.1 |
|  | BiLSTM | 142.5 | 145.6 | 148.7 | 151.4 | 150.3 | 149.6 | 148.5 | 151.9 | 150.4 | 152.9 |
|  | GRU | 175.2 | 179.3 | 174.5 | 178.9 | 180.6 | 181.6 | 170.5 | 171.6 | 177.2 | 178.6 |
|  | **En-GA** | 135.2 | 136.5 | 134.2 | 140.6 | 142.7 | 143.9 | 138.7 | 139.6 | 145.7 | 149.3 |
| **MAPE %** | LSTM | 0.59 | 0.58 | 0.60 | 0.59 | 0.61 | 0.62 | 0.59 | 0.60 | 0.58 | 0.62 |
|  | BiLSTM | 0.45 | 0.47 | 0.46 | 0.45 | 0.44 | 0.53 | 0.49 | 0.46 | 0.47 | 0.48 |
|  | GRU | 0.59 | 0.57 | 0.59 | 0.58 | 0.60 | 0.57 | 0.60 | 0.61 | 0.59 | 0.58 |
|  | **En-GA** | 0.42 | 0.41 | 0.39 | 0.42 | 0.40 | 0.41 | 0.45 | 0.43 | 0.39 | 0.44 |
| **MAE** | LSTM | 142.3 | 144.5 | 143.1 | 145.6 | 148.2 | 140.7 | 143.9 | 140.9 | 144.5 | 147.6 |
|  | BiLSTM | 109.6 | 109.8 | 110.2 | 112.4 | 108.6 | 111.3 | 115.2 | 112.5 | 116.3 | 113.2 |
|  | GRU | 140.3 | 138.9 | 142.7 | 139.6 | 141.5 | 142.7 | 144.5 | 143.2 | 140.8 | 141.3 |
|  | **En-GA** | 105.9 | 102.3 | 106.4 | 104.7 | 103.2 | 101.9 | 108.9 | 104.2 | 107.1 | 106.9 |

2. **Statistical Analysis:** We have also applied the Wilcoxon Signed Rank Test on the obtained results from these repeated simulations. This statistical analysis helps us make stronger and more reliable conclusions.

To determine if the differences in performance metrics across different runs were statistically significant, we applied the Wilcoxon Signed Rank Test. The results are summarized in Table 7.

**Table 7.** Wilcoxon signed rank test results

| Comparison | Statistic | p-value |
| --- | --- | --- |
| Ensemble vs LSTM | 0.0 | 0.001953125 |
| Ensemble vs BiLSTM | 0.0 | 0.001953125 |
| Ensemble vs GRU | 0.0 | 0.001953125 |

*+The p-values indicate whether there is a significant difference between the models' performances over multiple runs. A p-value less than 0.05 typically indicates a significant difference. In this case, the p-values for all comparisons (Ensemble vs. LSTM, Ensemble vs. BiLSTM, and Ensemble vs. GRU) across all metrics (MSE, MAE, RMSE, MAPE) are 0.001953125, which is significantly less than the 0.05 threshold. This demonstrates that the ensemble model consistently shows a statistically significant improvement in performance over the individual models.*

These additions have also been incorporated into our method section (**Section 3.4, page no. 10**), and the results have been updated accordingly in result and discussion section **(Section 6, page no, 19-20)**.

**Response to the Reviewer #1**

**1. Comment:** *Check for grammar and puntuation*

**Response:** We have thoroughly reviewed the manuscript and corrected all grammar and punctuation errors.

**2. Comment:** *101 ARIMA is written twice*

**Response:** We have corrected this duplication and ensured ARIMA is mentioned only once on line 101.

**3. Comment:** *number the equations*

**Response:** All equations have been numbered sequentially from Equation (1) to Equation (29) and appropriately cited in the text..

**4. Comment:** *181. what does se meams.*

**Response:** 'se' stands for 'series.' This was a typographical error, and it has been corrected to 'series' on line 181.

**5. Comment:** *221. define U*

**Response:** We have defined 𝑈 in the equation as follows:

- Forget Gate $\left( Ƒ_{t} \right)$: The forget gate is responsible for determining the retention or discarding of information from the previous cell state $\left( C_{t-1} \right)$ eq (1)

$Ƒ_{t}$= ⏀($w_{t}$ × $x_{t}$ + $U_{f} \times$ $h_{t-1}$ + $b_{f}$)	(1)

where w and b are weights and biases and ⏀ represent activation function, which is sigmoid in our study. $U_{f}$ is a weight mateix associated with the forget gate.

**6. Comment:** *verify the results in table 2.*

**Response:** Yes, we have verified the values in Table 2. We confirm that the results are accurate. The values differ because Table 2 presents the results for the entire dataset, while Table 4 further splits the validation data into four parts to assess performance on four different subsets. As a result, the MAPE, MAE, and RMSE scores vary between these two tables for all models due to the difference in dataset sizes, with the entire dataset containing more values and the split datasets having comparatively fewer values.

**7. Comment:** *verify the results in table 4.*

**Response:** Yes, we have also verified the values in Table 4. We confirm that the results are accurate. The values differ because Table 4 further splits the validation data into four parts to assess performance on four different subsets, whereas Table 2 presents the results for the entire dataset. This difference in dataset sizes leads to variations in the MAPE, MAE, and RMSE scores for all models, with the entire dataset containing more values and the split datasets having comparatively fewer values.

**Response to the Reviewer #2**

**1. Comment:** *Mention the objectives in the abstract.*

**Response:** Thank you for your valuable feedback. We have revised the abstract to explicitly include the objectives of our study.

**2. Comment:** *How does GA based feature selection contribute to the performance of the energy demand forecasting model?*

**Response:** Thank you for your insightful question. The Genetic Algorithm (GA)-based feature selection significantly improves the performance of the energy demand forecasting model by optimizing the selection of relevant features.

*+The GA identifies the best subset of features from the dataset, which includes historical energy consumption, weather variables, and temporal features, by exploring various combinations to maximize predictive accuracy. By choosing only the most relevant features, the GA reduces redundancy and noise, resulting in more accurate and robust model training. This optimized feature set enhances the model's performance, allowing it to generalize better from training data to unseen data, as shown by improved RMSE, MAPE, and MAE metrics. Furthermore, reducing the number of features decreases computational complexity, leading to faster training times without sacrificing accuracy.*

In our manuscript, we have detailed the application of GA-based feature selection in the Methods section (Section 3.2) and discussed its impact on model performance in the Results section (Section 6). Specifically, we noted that the GA-optimized feature subset led to a reduction in RMSE and MAPE, indicating a significant improvement in forecasting accuracy.

**3. Comment:** *Elaborate the types of deep learning models that are integrated into the ensemble framework proposed in the paper?*

**Response:** Thank you for your comment. We have provided detailed descriptions of the deep learning models integrated into the ensemble framework in Sections 3.3.1, 3.3.2, and 3.3.3 of the manuscript. For clarity and emphasis, we summarize the description here as well:

*The ensemble framework proposed in our paper integrates three types of deep learning models: Long Short-Term Memory (LSTM), Bi-directional Long Short-Term Memory (BiLSTM), and Gated Recurrent Unit (GRU). Each of these models is selected for its unique strengths in handling time series data. LSTM is adept at capturing long-term dependencies, BiLSTM leverages information from both past and future time points by processing data in both forward and backward directions, and GRU offers computational efficiency and reduces the risk of overfitting with its simpler architecture. These models are combined using a stacking ensemble technique to enhance the overall predictive performance of the energy demand forecasting model.*

The detailed descriptions can be found in the following sections of our manuscript:

- Section 3.3.1: Long Short-Term Memory (LSTM)
- Section 3.3.2: Bi-directional Long Short-Term Memory (BiLSTM)
- Section 3.3.3: Gated Recurrent Unit (GRU)

We hope this response clarifies the integration and roles of the deep learning models within our proposed ensemble framework.

**4. Comment:** *All the equations must be numbered and cited in the text.*

**Response:** All equations have been numbered sequentially from Equation (1) to Equation (29) and appropriately cited in the text.

**5. Comment:** *Describe the process of feature selection in details.*

**Response:** Thank you for your valuable feedback. We have revised Section 3.2 of the manuscript to provide a more detailed explanation of the Genetic Algorithm (GA) process for feature selection. Specifically, we have elaborated on the following aspects:

- The representation of chromosomes and the initialization of the population.
- The evaluation of fitness using the Mean Squared Error (MSE) metric.
- The selection, crossover, and mutation operations.
- The termination criteria for the GA process.
- The use of the DEAP (Distributed Evolutionary Algorithms in Python) library to implement the GA framework.

We have also included a detailed description of each step involved in the GA process, as illustrated in Figure 4 (**page no. 7**) of the manuscript.

**6. Comment:** *What are the key challenges addressed by using the proposed method?*

**Response:** We appreciate your valuable feedback. In response to this, we have included a new subsection (**Section 2.3**) titled **“Key Challenges Addressed by the Proposed Method”** in the manuscript (**page no. 5**). This subsection provides a detailed explanation of the main challenges in energy demand forecasting and how our proposed method addresses each of these challenges. Specifically, it discusses:

- The complexity of feature selection and how the GA efficiently identifies the optimal subset of features.
- Ensuring model generalization and avoiding overfitting by selecting the most informative features.
- Improving computational efficiency by reducing the number of features, thereby decreasing training time.

**7. Comment:** *Discuss the implications and potential applications of the ensemble deep learning framework in real-world energy demand forecasting scenarios.*

**Response:** Thank you for insightful comment. Based on your feedback, we have included a new subsection (**Section 6.3**) (**page no. 20**) titled “**Implications and Potential Applications' in the manuscript.**” This section discusses the practical implications and real-world applications of our ensemble deep learning framework for energy demand forecasting. Specifically, it highlights:

- Enhanced forecasting accuracy due to the integration of multiple deep learning models.
- Adaptability to different forecasting horizons (short-term, medium-term, and long-term).
- Efficient handling of complex and high-dimensional data through Genetic Algorithm-based feature selection.
- Implementation in smart grid systems for real-time energy demand forecasting and improved energy distribution efficiency.

**We extend our heartfelt gratitude to the editor and reviewers for their thorough review and insightful comments. Your constructive feedback has been invaluable in enhancing the quality of our manuscript. Thank you for your time and effort in helping us improve our work.**
