# Supplementary material for: An ensemble deep learning framework for energy demand forecasting using genetic algorithm-based feature selection
Source: PLoS One. 2025 Jan 15;20(1):e0310465. doi: 10.1371/journal.pone.0310465 (PMC11734974; doi:10.1371/journal.pone.0310465)
Supplement: Attachment — Submitted filename: Response to Reviewers.docx [file pone.0310465.s002.docx]

Replies to Concerns of Editor and Reviewers

**An Ensemble Deep Learning Framework for Energy Demand Forecasting Using Genetic Algorithm-Based Feature Selection**

PONE-D-24-12727

Dear Editors and Reviewers,

We would like to express our sincere gratitude to the editors and reviewers for their thorough examination and insightful feedback on our manuscript.

In response to the reviews, we have thoroughly revised our manuscript, taking into careful consideration every comment and suggestion provided. **Revised portions are highlighted in the manuscript in yellow.** The main corrections in the paper and the responses to the reviewer's comments are listed below.

**Response to the Editor**

**Comment:** *The authors are suggested to carefully go through each sentence of the manuscript and improve the linguistic quality of the manuscript.*

**Response:** In response to the editor's suggestion, we enlisted the help of a native English speaker to thoroughly revise the manuscript. We carefully reviewed each sentence and made several revisions to enhance its linguistic quality. The changes included both minor adjustments and more substantial edits.

First, we focused on punctuation and formatting, ensuring consistency and clarity throughout the text. This involved adding or removing spaces before and after punctuation marks, as well as making necessary adjustments to commas, periods, and other punctuation for improved readability.

Second, we refined the wording of numerous sentences and phrases to improve clarity and correct grammatical errors. For example, we revised sentences to ensure they conveyed the intended meaning more effectively and corrected any awkward phrasing or linguistic inaccuracies.

Additionally, we made structural edits to some parts of the manuscript. This involved reorganizing certain sentences or sections to improve the overall flow and coherence of the text. Furthermore, we removed redundant lines and unnecessary words to streamline the content and eliminate any repetition. This helped to sharpen the focus of the manuscript and enhance its overall readability.

**Journal’s Requirements**

In compliance with the journal's guidelines, we have thoroughly reviewed the reference list to ensure its completeness and accuracy. As part of this process, we carefully checked for any retracted papers and removed them where necessary. Additionally, we verified the DOI information for all references and have added the DOI to any entries where it was previously missing. All necessary corrections and updates to the references have been made to ensure they meet the required standards and align with the journal's requirements. The references that were corrected are highlighted in yellow and are presented below

*[4]	G. Franco and A. H. Sanstad, “Climate change and electricity demand in California,” Clim Change, vol. 87, no. 1, pp. 139–151, 2008, doi: 10.1007/s10584-007-9364-y.*

*[5]	S. Noureen, S. Atique, V. Roy, and S. Bayne, “Analysis and application of seasonal ARIMA model in Energy Demand Forecasting: A case study of small scale agricultural load,” Midwest Symposium on Circuits and Systems, vol. 2019-Augus, pp. 521–524, 2019, doi: 10.1109/MWSCAS.2019.8885349.*

*[11]	M. Q. Raza and A. Khosravi, “A review on artificial intelligence based load demand forecasting techniques for smart grid and buildings,” Renewable and Sustainable Energy Reviews, vol. 50, pp. 1352–1372, 2015, doi: 10.1016/j.rser.2015.04.065.*

*[22]	Z. Ullah, F. Al-Turjman, L. Mostarda, and R. Gagliardi, “Applications of Artificial Intelligence and Machine learning in smart cities,” Comput Commun, vol. 154, pp. 313–323, 2020, doi: 10.1016/j.comcom.2020.02.069.*

*[23]	W. Xu, H. Peng, X. Zeng, F. Zhou, X. Tian, and X. Peng, “A hybrid modelling method for time series forecasting based on a linear regression model and deep learning,” Applied Intelligence, vol. 49, no. 8, pp. 3002–3015, 2019, doi: 10.1007/s10489-019-01426-3.*

*[24]	F. Mohammad and Y. C. Kim, “Energy load forecasting model based on deep neural networks for smart grids,” International Journal of System Assurance Engineering and Management, vol. 11, no. 4, pp. 824–834, 2020, doi: 10.1007/s13198-019-00884-9.*

*[25]	T. Ahmad, H. Zhang, and B. Yan, “A review on renewable energy and electricity requirement forecasting models for smart grid and buildings,” Sustain Cities Soc, vol. 55, p. 102052, 2020. doi: 10.1016/j.scs.2020.102052.*

*[26]	N. Al-Taleb and N. A. Saqib, “Towards a hybrid machine learning model for intelligent cyber threat identification in smart city environments,” Applied Sciences, vol. 12, no. 4, p. 1863, 2022. doi:* [*10.3390/app12041863*](https://doi.org/10.3390/app12041863)

*[29]	A. Kulshrestha, V. Krishnaswamy, and M. Sharma, “Bayesian BILSTM approach for tourism demand forecasting,” Ann Tour Res, vol. 83, p. 102925, 2020. doi: 10.1016/j.annals.2020.102925.*

*[39]	L. Rice, E. Wong, and J. Z. Kolter, “Overfitting in adversarially robust deep learning,” 37th International Conference on Machine Learning, ICML 2020, vol. PartF16814, pp. 8049–8074, 2020.*

*[40]	P. J. Brockwell and R. A. Davis, “Introduction to Time Series and Forecasting - Second Edition,” Springer-Verlag, p. 449, 2002. doi: 10.1007/978-3-319-29854-2*

*[47]	R. Leardi, R. Boggia, and M. Terrile, “Genetic algorithms as a strategy for feature selection,” J Chemom, vol. 6, no. 5, pp. 267–281, 1992. doi:* [*10.1002/cem.1180060506*](https://doi.org/10.1002/cem.1180060506)

**We extend our heartfelt gratitude to the editor and reviewers for their thorough review and insightful comments. Your constructive feedback has been invaluable in enhancing the quality of our manuscript. Thank you for your time and effort in helping us improve our work.**
